# Mundane? Demographic characteristics as predictors of enrolment onto the National Health Insurance Scheme in two districts of Ghana

**DOI:** 10.1186/s12913-018-3155-1

**Published:** 2018-05-04

**Authors:** Anthony Seddoh, Fuseini Sataru

**Affiliations:** International Finance Corporation, Independence Ave. Ridge, CT 2638 Accra, Ghana

**Keywords:** National Health Insurance Scheme, Historical context of health care financing in Ghana, Social protection, Enrolment

## Abstract

**Background:**

In 2003, Ghana passed a law to establish a National Health Insurance Scheme (NHIS) to serve as the main vehicle for achieving universal health coverage. Over 60% of the population had registered by 2009. Current active membership is however 40%. The stagnation in growth has been recorded across all the membership categories. Clearly, the Scheme is falling short of its core objective. This analysis is a critical thematic contextual examination of the effects of demographic factors on enrolment onto the Scheme.

**Methods:**

Demographic secondary data for 625 respondents collected (using a structured questionnaire) during a cross-sectional household survey in an urban, Ashaiman, and rural, Adaklu, districts was analyzed in univariate and multivariate logistic regression models using Statistical Package for Social Scientists (SPSS). Statistical significance was set at *P*-value < 0.05. Variables included in the analysis were age, gender, education, occupation and knowledge about the NHIS.

**Results:**

Seventy-nine percent of the survey respondents have ever enrolled onto the NHIS with three-fifths being females. Of the ever enrolled, 63% had valid cards. Age, gender and educational level were significant predictors of enrolment in the multivariate analysis. Respondents between the ages 41–60 years were twice (*p* = 0.05) more likely to be enrolled onto a district Scheme compared with respondents between the ages 21–40 years. Females were thrice (*p* = 0.00) more likely to enroll compared with males. Respondents educated to the tertiary, five times (*p* = 0.02), and post-graduate, four times (*p* = 0.05), levels were more likely to enroll compared with non-educated respondents. No significant association was observed between occupation and enrolment.

**Conclusion:**

Uptake of the scheme is declining despite high awareness and knowledge. Leadership, innovation and collaboration are required at the district Scheme level to curtail issues of low self-enrolment and to grow membership. Otherwise, the goal of universal coverage under the NHIS will become merely a slogan and equity in financial access to health care for all Ghanaians will remain elusive.

## Background

Health care financing, constantly, has been in transition in many countries, especially the developing ones [1]. Country level health care financing mechanisms are now no longer only a matter of national level policy discourse and concerns, but have transcended into international development agendas as manifested in the Sustainable Development Goals (SDGs), and the Universal Health Coverage and Global Health movements [2–4]. Like most countries seeking to ensure the well-being of its citizens, Ghana has experimented with and introduced different models of health care financing. The National Health Insurance Scheme (NHIS) was introduced in 2003 as a social intervention programme on health through the passage of the National Health Insurance law, Act 650 [5–8]. Embedded in the Scheme’s design are universal coverage assurance and equity in financial access goals [9–13].

Amended in 2012, as Act 852, the law now consolidated the previously semi-autonomous District-wide Mutual Health Insurance Schemes (DMHIS) under the centralized National Health Insurance Authority (NHIA). The preparations phase and integration of the Scheme into the country’s health system took two years and access to care under the Scheme commenced in 2005. Benefits accruing to enrollees include medicines (mainly core drugs and specified in a list) and care services (both in-patient and out-patient) that has been estimated to cover 95% of all disease conditions in the country [6, 7].

The NHIS law defines two types of memberships, premium and non-premium paying. In a move towards fulfilling its core mandate, the NHIS law under Legislative Instrument 1809 exempts from premium payment a large segment of the population. Some of the exempted are however required to pay a registration fee [6, 7]. The non-compliance and non-enforcement of the obligatory subscription onto an insurance scheme in Ghana is attributed to the socially driven nature of the NHIS law. In accordance with the tenets of the law, the operations of the NHIA and the affiliate DMHISs have mainly been focused on protection of the vulnerable and poor by ensuring access to the defined benefits package [14–16]. Forty percent of residents in the country are actively enrolled. The current active membership is reflective of this exemptions policy with 66.9% classified as premium exempted [17].

**Fig. 1:**
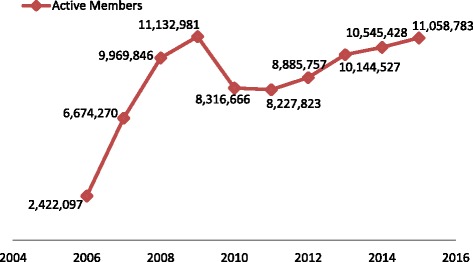
Trends in active membership of the Ghana NHIS

Membership of the Scheme grew exponentially in the early years of operations (Fig. 1). The reversal in growth from 2009 to 2011 was largely due to the accumulated severe debt the NHIA had owed service providers – in some cases up to nine months of unpaid claims. Service delivery had been disturbed making it harder ensuring services were delivered to members with some facilities turning away NHIS card holders [15, 18, 19]. This resulted in low demand for the NHIS across all the membership categories [11–16]. The situation has however been partially remedied through a mix of strategies that include; re-structuring of the Scheme and developing both human and technical competencies; review of the provider payment systems and terms; introduction of technology in registration and card processing; and re-strategizing from centralized registration at DMHIS offices to mass mobile registration centers especially in rural areas [17, 19]. That notwithstanding, uptake and coverage remain disproportionately low [13–15]. For instance, active membership grew by 4.9%, representing a 2.5% increase in coverage, between 2014 and 2015.

Overall, several factors - Scheme, health system and population related - account for the slow growth in membership [15, 18, 20]. Whereas some of these factors are salient, others are less visible thereby requiring critical thematic and contextual exploration. The research on factors of enrolment onto insurance schemes in sub-Saharan Africa, not least Ghana, is largely skewed towards socio-economic, health system and scheme related factors [1]. Even in literature search, we did not come across a study that focused exclusively on quantifying the effects of demographic factors on enrolment onto the Ghana NHIS. In light of this, this study examined the effects of demographic factors on enrolment in two districts of Ghana.

## Methods

### Study setting

Ashaiman is a clustered urban municipality located in the Greater Accra Region with a heterogeneous population of 190,972. There are 49,936 households in the district averaging 3.7 persons per household. Majority (31.9%) of the district’s population is between 0 and 14 years. Ninety two percent of residents in the municipality are engaged in some form of income earning activity with 36.5% into sales and service businesses. Seventeen percent of mortality in the municipality is due to accidents, homicide and related causes (16.7%) [21]. Four percent of the municipality’s population is classified as poor [22] and 58% had valid NHIS cards at the time of the study [23].

Adaklu District, located in the Volta Region, has a comparatively smaller population of 36,391 with a slightly higher proportion of females, 51%. There are 6089 households in the district averaging six persons per household. Majority, 36.4%, of the district’s population is below 15 years and 7.6% are above 60 years. Ninety-five percent of the economically active population of the district is self-employed of which 63.1% are into skilled agriculture, forestry and fishery business [24]. Poverty (89.7%) is endemic in this district [21]. Valid enrolment at the time of the study onto the NHIS was 66.8% of the districts population [23].

### Sampling and data collection

Secondary data analyzed in this paper were collected from 19th January to 5th February 2016 as part of a household baseline cross-sectional survey conducted by the African Health Markets for Equity (AHME) project, 2013–2018, to identify and (re)enrol extremely poor Ghanaians onto the NHIS in ten pilot districts. Only data from Ashaiman and Adaklu districts are made available for this analysis by the project. Communities were randomly selected for the survey. Distance, nearness or otherwise to an NHIS registration centre and an NHIS accredited health facility, were factors guiding the inclusion of communities in the survey. Fifteen communities participated in the survey, five in Ashaiman and ten in Adaklu district. In the Ashaiman municipality, household sampling interval was ten whereas in Adaklu district the sampling interval was four. Survey respondents were randomly recruited based on age (18 years and above) and being the head or nominated by the household to respond. A structured questionnaire was administered. This questionnaire was an adaptation of the Common Targeting Mechanism tool – first pretested in 2009 and currently still in pilot phase – developed by the Ministry of Gender, Children and Social Protection for social intervention programmes in the country. Data was collected on respondents’ socio-demographic information, knowledge about the NHIS, membership and experiences with the NHIS registration process and experiences with use of the card to access health care. Findings for the latter three thematic research areas are presented in another paper. A total of 625 interviews were conducted, 309 in Ashaiman municipality and 316 in Adaklu district. All data collectors were experienced social scientists, who were fluent in at least two of the dominant languages spoken in the study settings.

### Statistical analysis

Variables included in the analysis were age, gender, education and occupation. Knowledge about the existence of the NHIS was also included as it directly related to the objectives of this analysis. In the first stage, data for the 625 respondents was uploaded onto Statistical Package for Social Sciences (SPSS), version 20, for a univariate logistic regression analysis. The second stage involved adjusting for confounders in a multivariate analysis to determine the significant predictors. Variables that were not significant in the univariate model were not included in the multivariate analysis. We set statistical significance at *P*-value < 0.05. Results of the analysis are presented as Odds Ratios with their accompanying *P*-Values and Confidence Intervals. A frequency analysis was also done for NHIS membership status and demographic characteristics of survey respondents.

## Results

Fifty-six percent of the survey respondents were female. The age distribution shows majority (56.6%) were between the ages 21–40 years. Thirty percent of survey respondents were educated to the Junior High School level. Trading (26.7%), was the predominant occupation. Seventy-nine percent of the survey respondents have ever enrolled onto the NHIS of which three-fifth were females. Sixty-three percent of the ever enrolled had valid cards of which 51.5% were seen by the research team. A disaggregation of membership by districts showed a marginally higher ever enrolled membership in Ashaiman municipality (79.6%) than Adaklu district (78.2%). Valid membership was however substantially higher in Ashaiman municipality (71.1%) than Adaklu district (53.8%).

Results of the univariate regression analysis are presented in Table 1. Education, above the primary level, was significantly associated with enrolment, likewise females (*p* = 0.00) and respondents who were 60 years and above (*p* = 0.03). Table 2 shows findings from the multivariate analysis. Respondents between the ages 41–60 years were twice (*p* = 0.05) more likely to be enrolled onto a district scheme compared with respondents between the ages 21–40 years. Females were thrice (*p* = 0.00) more likely to enroll compared with males. Further, respondents educated to the tertiary level, five times (*p* = 0.02) and post-graduate, four times (*p* = 0.05) levels were more likely to enroll compared with non-educated respondents. No significant association was observed between occupation and enrolment.

**Table 1:** Univariate model of demographic predictors of enrolment onto the NHIS

Variable	N (%)	P -value	Unadjusted OR	95% CI
Age group (years)				
Below 20	26(26.9)	0.64	0.8	0.33–1.98
21–40 (Ref)	352(56.6)	1		
41–60	176(28.3)	0.22	1.33	0.84–2.11
61 and above	68(10.9)	0.03	2.46	1.08–5.59
Gender				
Male (Ref)	272(43.5)	1		
Female	353(56.5)	0.00	2.1	1.41–3.12
Education				
None (Ref)	166(26.9)	1		
Primary	100(16.2)	0.13	1.59	0.87–2.88
JSS	187(30.3)	0.03	1.72	1.04–2.84
SHS	93(15.1)	0.08	1.75	0.93–3.28
Tertiary	32(5.2)	0.03	3.83	1.11–13.20
Postgraduate	39(6.3)	0.025	3.47	1.18–10.31
Read				
Yes	381(62.2)	0.027	1.572	1.05–2.35
No (Ref)	232(37.8)	1		
Write				
Yes	357(58.3)	0.16	1.33	0.90–1.99
No (Ref)	255(41.7)	1		
Occupation				
Trader	166(26.7)	0.36	1.37	0.70–2.68
labourer	103(16.6)	0.79	0.91	0.40–1.83
civil servant	33(5.3)	0.17	2.47	0.68–8.99
farming/agric	67(10.8)	0.43	1.41	0.60–3.28
Craft and related bus.	97(15.6)	0.2	0.64	0.332–1.26
others	62(10.0)	0.11	0.56	0.26–1.15
unemployed (Ref)	93(15.0)	1		
Ever heard of NHIS				
Yes	609(98.1)	0.048	3.37	1.01–11.22
No (Ref)	12(1.9)	1

**Table 2:** Factors (adjusted) determining NHIS enrolment

Variables	N (%)	*P* value	Adjusted OR	95% CI
Age group (years)				
Below 20	26(26.9)	0.86	0.91	0.33–2.49
21–40 (Ref)	352(56.6)	1		
41–60	176(28.3)	0.05	1.67	0.99–2.79
61 and above	68(10.9)	0.16	2.97	1.23–7.16
Gender				
Male (Ref)	272(43.5)	1		
Female	353(56.5)	0.00	2.73	1.76–4.24
Education				
None (Ref)	166(26.9)	1		
Primary	100(16.2)	0.08	1.88	0.94–3.79
JSS	187(30.3)	0.10	1.96	0.88–4.35
SHS	93(15.1)	0.11	2.13	0.84–5.44
Tertiary	32(5.2)	0.02	5.26	1.28–21.58
Postgraduate	39(6.3)	0.05	4.14	1.02–16.85
Read				
Yes	381(62.2)	0.56	1.23	0.62–2.46
No (Ref)	232(37.8)	1		
Ever heard of NHIS				
Yes	609(98.1)	0.21	2.29	0.63–8.32
No (Ref)	12(1.9)	1		

## Discussion

Health care financing in Ghana has evolved initially as a cost sharing system which subsequently transitioned into full-cost-recovery [25, 26]. At introduction, the NHIS was acclaimed as a game changer. However, twelve years on, enrolment has not reached the level envisioned [2, 15, 27]. This is irrespective of it being a government issued health insurance backed by an extensive exemptions list and, supposedly, affordable premium rates [14]. This analysis examined the effects of demographic factors on enrolment onto the Ghana NHIS. Sixty-three percent of the survey respondents were valid NHIS card holders and this is substantially higher than the national average of 40%. Valid memberships were 71.1% for Ashaiman municipality and 53.8% for Adaklu district.

Earlier exploratory studies found a lack of knowledge and access to information as adversely affecting enrolment [11, 28, 29]. Our observations of high enrolment in these districts may be attributable to an increase in awareness on the Scheme and health system related factors. The importance of collaborating with and indulging community level stakeholders by DMHISs to grow membership of the Scheme may also be a contributing factor. Working with and through community based organizations, such as traders associations, religious, women and youth groups, and community interest agencies, such as Social Welfare and Information Services Departments, did not only widen the coverage of information about the Scheme. It also served to improve the dearth of penetration of such messages. Such partnerships also provided platforms for DHIMSs to efficiently and conveniently implement their communication and registration programmes. District schemes also maximized their synergistic relationships with the health care system to improve their operations. Aligning their communication programs on the benefits of acquiring a health insurance brought about much needed uniformity, consistency and better assimilation of the messages as communities easily identify with health workers. This, further, had the potential of bringing about improvements in the quality of care offered to card holders, seeing that quality of care has been documented in the literature as a determinant of (re)enrolment onto the NHIS in Ghana [3, 14, 15].

The analysis also showed a higher enrolment among females. Whereas males and young people are generally more risk averse and exhibit indifferent behaviours in health related matters, women and the elderly are more self-health conscious thereby planning for it and in this case by acquiring a health insurance card [3, 9, 30]. Higher enrolment on the basis of gender and age may also be explained by the free membership concessions granted to pregnant women, persons above 70 years and the extremely poor – poverty has gender connotations in Ghana [31, 32]. We found that persons above 40 years had a higher probability to enroll. While this may be suggestive of adverse selection onto the Scheme, it more importantly is also indicative of a bridging of the gap in financial accessibility to health care for vulnerable groups. The implication may be that as a social protection intervention, the NHIS is achieving its goal of promoting equity in access [9, 11, 12]. We were however not able to comment on whether the vulnerable and poor were actually being covered given the broad sweep approach. For this to be determined purpose targeting using enrolment strategies such proxy-means and geographic targeting approaches to identify and enrol more vulnerable groups onto the Scheme needs to be considered [33–36].

Education was also a positive predictor of enrolment onto the NHIS in our analysis. Education comes with knowledge, higher exposure and access to information which critically facilitates and translates into informed decisions to enroll [29, 37]. Following, the significant association observed between higher educational attainment and enrolment may be inferred to mean the NHIS is attractive to all the different social classes within the population. The implication however is that DHMISs will have to undertake aggressive marketing and registration outreach programmes targeting the uneducated [32, 38]. Education as a strong predictor of enrolment was also confirmed by earlier findings by Gobah and Liang (2011) and John (2013) in Ghana and Kirigia et al. (2005) in South Africa.

Our analysis has some limitations. It is narrow in scope in relation to the variables and our chosen area of focus. First, the absence of data on other demographic variables such as marital status and family size among others may have marginally over-estimated the associations we observed in the analysis [30]. Secondly, limiting our analysis to only demographic factors may have masked the joint effects of other demand and supply side related factors on (re)enrolment and the reasons explaining the decisions on enrolment [12]. Thirdly, we caution that associations between the variables analyzed and likelihood of (re)enrolment should not be inferred as reasons for non-enrolment among survey respondents that did not enroll or had expired cards. We, however, note that our analysis was rigorous hence the findings are applicable in the study settings. The findings also provide useful insight on how individual personal attributes contribute, unconsciously, in enrolment onto an insurance scheme.

## Conclusions

Uptake of the Scheme is declining despite high awareness and knowledge. This analysis reports age, gender and educational status as significant determinants of enrolment onto the NHIS in an urban and rural district of Ghana. This calls for introspection into the operations of the NHIS. Regular updates and tailored communication programs are important in generating and sustaining interest in the Scheme. District schemes should develop and maintain partnerships with community based groups and other local agencies engaged in health to socially market health insurance where they exist. The goal of universal coverage and equity in access to health care under the NHIS remains attainable, but will require innovation, stakeholder engagement and concerted effort on the part of DMHISs to achieve.

## Abbreviations

AHME: African Health Markets for Equity
DMHIS: District-wide Mutual Health Insurance Scheme
NHIA: National Health Insurance Authority
NHIS: National Health Insurance Scheme
